# Potential usefulness of apolipoprotein A2 isoforms for screening and risk stratification of pancreatic cancer

**DOI:** 10.2217/bmm-2016-0209

**Published:** 2016-09-27

**Authors:** Kazufumi Honda, Sudhir Srivastava

**Affiliations:** 1 Division of Chemotherapy & Clinical Research, National Cancer Center Research Institute, Tokyo 104–0045, Japan; 2 Japan Agency for Medical Research & Development (AMED) CREST, Tokyo 100–0004, Japan; 3 Division of Cancer Prevention, National Cancer Institute, Rockville, MD 20852, USA

**Keywords:** apolipoprotein A2 isoforms, early detection for pancreatic cancer, National Cancer Institute Early Detection Research Network, NCI EDRN, plasma/serum biomarker

## Abstract

Given the low incidence of pancreatic cancer in the general population, screening of pancreatic cancer in the general population using invasive modalities is not feasible. Combination of invasive screening with noninvasive biomarkers for pancreatic cancer and its precancerous lesions has the potential to reduce mortality due to pancreatic cancer. In this review, we focus on biomarkers found in the blood that can indicate early-stage pancreatic cancer, and we discuss current strategies for screening for pancreatic cancer. We recently identified a unique alteration in apolipoprotein A2 isoforms in pancreatic cancer and its precancerous lesions, and we describe its clinical usefulness as a potential biomarker for the early detection and risk stratification of pancreatic cancer.

Pancreatic cancer is the fourth leading cause of cancer in Japan and the USA, and has the worst prognosis of solid tumors [1,2]. The Japanese Association of Clinical Cancer Center reported that the 5- and 10-year survival rates for pancreatic cancer are 6.3 and 4.9%, respectively [3]. Early detection of pancreatic cancer is very difficult, because the pancreas is located deep within the abdominal cavity, and patients with early-stage pancreatic cancer do not present unique clinical symptoms [4]. Symptoms of abdominal pain, jaundice and/or weight loss are first reported in locally advanced or metastatic pancreatic cancer [5]; however, those symptoms are not unique to pancreatic cancer [6,7]. The majority of patients with pancreatic cancer consist of either metastatic or locally advanced pancreatic cancer without unique clinical symptoms, and 80% of these patients present with advanced stages of pancreatic cancer [8,9].

Pancreatic ductal adenocarcinoma (PDAC) is the predominant histological type of pancreatic cancer. Surgical resection is the only potentially curative therapy for PDAC. The locally advanced or metastatic lesions of PDAC complicate the curative surgery for PDAC. Approximately 20% of patients with PDAC present with potentially curative disease, such as resectable or borderline resectable cancers [8]. A recent study has suggested that it takes at least 15 years from the initial mutation to the development of metastatic pancreatic cancer [10]. This finding indicates that there is a large window of opportunity for the early diagnosis of PDAC, even though the vast majority of patients with PDAC present with locally advanced and metastatic pancreatic cancer.

Since there is a relatively low incidence of pancreatic cancer in the general population, screening of unselected populations for asymptomatic pancreatic cancer is not cost-effective with current technologies [8]. There are a variety of factors that influence pancreatic cancer risk. For example, approximately 1% of adults older than 50 years of age who have new-onset diabetes are expected to develop pancreatic cancer within 3 years [11–13]. In addition, family history of pancreatic cancer [14–18], environmental factors such as smoking [19] and factors related to dietary habits, including BMI [20,21], red meat intake [22], low fruit and vegetables intake [22], diabetes [23] and alcohol intake [5,24], as well as hereditary pancreatitis syndrome [25] and certain genotypes have a certain risk of pancreatic cancer [7,26–28]. Particularly in terms of screening and treatment costs, groups with a high risk of pancreatic cancer would likely benefit from early detection.

As detection methods are refined, imaging could also be applied to low-risk groups, especially if it were combined with an inexpensive screening biomarker such as a blood test.

In general, PDAC develops from three precancerous lesions: pancreatic intraepithelial neoplasias [29]; intraductal papillary mucinous neoplasms (IPMN) [30]; and mucinous cystic neoplasms (MCN) [31]. Currently there is a great need for the development of noninvasive biomarkers for the early detection of pancreatic cancer including of the precancerous lesions of pancreatic cancer (pancreatic intraepithelial neoplasias, IPMN with high-grade dysplasia or carcinoma *in situ*), in order to have an impact on survival benefit for pancreatic cancer. Techniques in current use for detecting early-stage pancreatic cancer are more invasive than blood tests and include endoscopic ultrasound (EUS) and magnetic resonance cholangiopancreatography (MRCP) or endoscopic retrograde cholangiopancreatography (ERCP).

Taking into account the high cost of screening for the incidence of pancreatic cancer, it has been considered that invasive strategies using an imaging modality should be performed for patients who have a relatively high risk of pancreatic cancer after screening for subjects who have a high risk of pancreatic cancer by a noninvasive strategy such as a blood test using biomarkers.

In this review, we focus on plasma/serum biomarkers for the detection of early stages of pancreatic cancer and its risk diseases such as precursors of pancreatic cancer, in order to decrease the mortality of pancreatic cancer.

We recently identified a potential plasma biomarker for the detection of patients with pancreatic cancer and of patients with diseases with a high risk of pancreatic cancer, and we reported the usefulness of this biomarker in a collaboration with the National Cancer Institute Early Detection Research Network (NCI EDRN) [32,33] and Japanese study group. This biomarker can distinguish patients with stage I/II of pancreatic cancer and its risk diseases from healthy subjects by detection of the cleaved pattern of the C-terminal ends of the amino acids of the apolipoprotein A2 (apoA2) homodimer [34–36].

We discuss the current strategies for the screening of pancreatic cancer. In addition, we describe the potential clinical usefulness of apoA2 isoforms for the detection of pancreatic cancer and its risk diseases.

## Biomarkers for pancreatic cancer detected by a blood test

### Existing conventional biomarkers for the detection of pancreatic cancer using a blood test

#### Carbohydrate antigen 19–9

Carbohydrate antigen 19–9 (CA19–9) is the most common biomarker that is used for monitoring the response to therapy in patients with pancreatic cancer and this biomarker has been approved by the US FDA as an *in vitro* diagnostics (IVD) [37]. The weak point of CA19–9 for the screening of pancreatic cancer is that CA19–9 mainly reacts with the advanced stage of pancreatic cancer, and it is not sensitive enough for the detection of early pancreatic cancer [38]. In addition, CA19–9 is not expressed in populations without the sialylated Lewis blood group antigen [39,40].

CA19–9 is not recommended for screening of pancreatic cancer in an asymptomatic population according to the American Society of Clinical Oncology [41]. Kim *et al.* reported that CA19–9 showed high sensitivity for pancreatic cancer. However, the positive predictive value was 0.9%, and they concluded that screening for pancreatic cancer in asymptomatic subjects using CA19–9 is ineffective, because of the low-positive predictive value for detection of pancreatic cancer [42].

### Carcinoembryonic antigen

Carcinoembryonic antigen (CEA) is the second most common serum biomarker used clinically to detect pancreatic cancer [37]. It was initially discovered as a biomarker for the detection of colorectal cancer. CEA reacts not only with colorectal cancer, but also with several other cancers such as pancreatic cancer. A recent compendium reported median CEA estimates of 54% sensitivity and 79% specificity in the detection of pancreatic cancer in an analysis of 13 studies reporting CEA values in a total of 1323 cases [43].

### Other current biomarkers for the detection of pancreatic cancer by a blood test

A large number of biomarkers that can be used to detect pancreatic cancer by a blood test have been reported in the last three decades including DUPAN-2 [44], Span-1 [45], NCC-ST-439 [46,47], osteopontin [48], macrophage inhibitory cytokine-1 [49], prolyl 4-hydroxylation of α-fibrinogen [50] and matrix metaroprotease-7 [51]. Each of those biomarkers detects pancreatic cancer in a unique way. Some of those biomarkers have already been applied as IVD products in a clinical setting. However, these biomarkers have not yet been used for screening of pancreatic cancer because their positive detection rates are not high enough for the early detection of pancreatic cancer.

Recently, a diagnostic model that was based on differences in the metabolomic profile between pancreatic diseases and healthy controls was reported for the detection of pancreatic cancer by Kobayashi *et al.* For distinguishing the early stage of pancreatic cancer, this model measures the peaks of four metabolites: 1,5-anhydro-d-glucitol, histidine, inositol and xylitol using GC–MS [52]. This model showed higher accuracy than conventional biomarkers in detecting patients with resectable pancreatic cancer; the sensitivity of this method for discrimination of patients with resectable pancreatic cancer was 77.8%, which was higher than that of CA19–9 or CEA even in their validation study.

More recently, Melo *et al.* described a new and exciting biomarker for the highly sensitive and specific diagnosis of pancreatic cancer by using exosomes carrying GPC1. The most impressive finding with GPC1 is the diagnosis of the early stage of pancreatic cancer from other benign pancreatic diseases, with 100% sensitivity and specificity [53]. GPC1 can serve as a potential biomarker for a noninvasive diagnostic and a screening tool for pancreatic cancer. This study is a pioneer study since GPC1 in exosomes offers better sensitivity and specificity than any other marker that is under evaluation for use in clinical practice. It is hoped that further studies aimed at clinical development to translate this technology to practicable clinical methods will be undertaken [54].

Not only protein- and glycol-based biomarkers but also DNA- and RNA-based biomarkers for the detection of pancreatic cancer have been reported. In particular, diagnostic panels that were constructed by combining expression information of multiple miRNAs in the blood for detection of the early stage of pancreatic cancer have gained considerable attention. Several potential diagnostic panels that showed impressive findings for early detection of pancreatic cancer have been reported [55]. Kojima *et al.* reported that the combination analysis of four miRNAs: miR-6075, miR-6799-5p, miR-125a-3p and miR-6836-3p, in the blood stream had a high AUC value (0.94) for distinguishing patients with pancreatic cancer from healthy controls. The AUC value of this model was higher than that of CA19-9 and CEA.

To apply these potential biomarkers to the clinical setting, detailed validation studies should be designed. The best way to confirm the claims of these interesting reports is to have independent validation studies performed by other research groups or by the NCI EDRN [56].

## Possible detection of the early stage of pancreatic cancer & of risk diseases by apoA2 isoforms

### Isolation of a biomarker for the early detection of pancreatic cancer by plasma protein profiles using top-down proteomics

To detect the early stage of pancreatic cancer using a noninvasive biomarker, we screened comprehensive expression profiles of proteins from plasma that was obtained from healthy volunteers and from patients with pancreatic cancers for a plasma biomarker, by using the technology of top-down proteomics. Top-down proteomics is a technique that can be used to perform a comprehensive analysis to obtain a protein expression profile using mass spectrometry (MS) without digesting the protein by a protease. It includes MALDI and SELDI-TOF-MS [57]. In 2005, we identified a diagnostic panel from a plasma protein profile that was constructed of four protein peaks and that could distinguish patients with PDAC from healthy subjects with high accuracy [34]. The AUC value of the diagnostic panel, which was calculated by receiver-operating characteristic analysis to distinguish PDAC from healthy controls, was 0.978. The algorithm to calculate the diagnostic panel was generalized from the protein expression profile of a training cohort (PDAC, n = 71; and healthy subject, n = 71) using an artificial intelligence technique. The diagnostic panel was independently confirmed by two other validation cohorts that were collected from two independent Japanese hospitals. Specifically, in that study we showed that the protein peak in the environs of 17.5 kDa in patients with PDCA was significantly decreased in comparison with healthy subjects [34].

Subsequently, in 2007, Ehmann *et al.* independently screened sera of PDCA and healthy controls that were collected by Heidelberg University Department of Surgery (Heidelberg, Germany) for a biomarker that could be used for development of an assay for the early detection of pancreatic cancer. By using SELDI-TOF-MS, they identified a biomarker that was significantly decreased in the sera of patients with PDCA compared with healthy controls. This protein also had a molecular weight (MW) of 17.5 kDa [58]. They identified this protein using bottom-up proteomics and MS as an apoA2 isoform in which an amino acid at the C-terminal end was lacking in an apoA2 homodimer. The AUC of this apoA2 isoform to distinguish PDAC from healthy controls was 0.94 according to Ehmann’s report [58]. In addition, in 2010, Xue *et al.* also identified an apoA2 isoform as a serum biomarker that was reduced in pancreatic cancer by using SELDI-TOF-MS. They also reported that a 17.5 kDa protein was reduced in the sera of patients with PDAC in comparison with healthy controls, and they confirmed that this protein was an apoA2 isoform [59]. Thus, the significant reduction in apoA2 in PDAC was independently validated by other study groups in Germany and Australia.

### Possible detection of PDAC & pancreatic disorders using the expression level of apoA2 as measured using MALDI-TOF-MS

**Figure 1.**
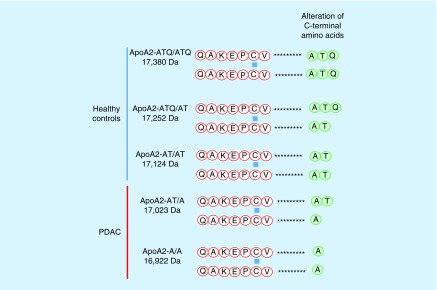
**Amino acid sequences of the apolipoprotein A2 isoforms** [36]. Apolipoprotein AII is comprised of 77 amino acids and forms a homodimer in humans via a disulfide bond involving Cys6. The theoretical molecular weights of the five isoforms of apolipoprotein A2 are shown at left [36].

The mature apoA2 protein consists of 77 amino acids with 1 cysteine residue at position 6, which represents the sulfhydryl group responsible for the formation of disulfide bridges. Most of the apoA2 in human blood is present as a disulfide-linked homodimer (96%) [58]. A small fraction forms heterodimers with apolipoprotein D and E, and another minor fraction is present as a monomer [60]. The data of our and other groups of analyses using MALDI-TOF-MS indicated that there are three isoforms of circulating apoA2 homodimers in the blood stream of healthy subjects [61,62]. Moreover, we newly identified another two isoforms from the plasma of patients with PDAC [35]. Figure 1 shows the C-terminal amino acids of the five isoforms of apoA2 homodimers. We named these isoforms as: apoA2-ATQ/ATQ (MW, 17,380 Da), apoA2-ATQ/AT (17,252 Da), apoA2-AT/AT (17,124 Da), apoA2-AT/A (17,023 Da) and apoA2-A/A (16,922 Da). The three apoA2 isoforms of apoA2-ATQ/ATQ, apoA2-ATQ/AT and apoA2-AT/AT were detected in healthy blood, and the novel two isoforms of apoA2-AT/A and apoA2-A/A were identified in the plasma of pancreatic cancer and risk diseases of pancreatic cancer in our MALDI-TOF-MS study. The concentration of each isoform of the apoA2 homodimer in the plasma can be semiquantitatively measured after its isolation, using information regarding the different MW of each isoform obtained by MALDI-TOF-MS analysis. In order to semiquantify the concentration of apoA2 isoforms in the plasma in a high-throughput format, we newly developed a sophisticated measurement method, the orthogonal MALDI-QqTOF (quadrupole TOF)-MS system [35].

To confirm the diagnostic accuracy of apoA2-ATQ/AT, we prospectively collected plasma samples of healthy controls, of PDAC, and of the risk diseases of pancreatic cancer and other gastroenterological malignancies from seven independent medical institutions in Japan (n = 833). We measured the expression level of apoA2-ATQ/AT in the plasma of patients with PDAC using MALDI-QTOF-MS. We found that the level of apoA2-ATQ/AT in any clinical stage of PDAC was significantly decreased in comparison with that of healthy controls. The AUC value of apoA2-ATQ/AT in pancreatic cancer to distinguish patients with PDAC from healthy controls was 0.876.

Moreover, we confirmed this result with the German cohort that was collected by Heidelberg University. The AUC value of apoA2-ATQ/AT in the German cohort was 0.958, and the diagnostic accuracy to distinguish PDAC from healthy controls was confirmed by the German cohort [35].

ApoA2 is a major apolipoprotein and is an important component of high-density lipoprotein. ApoA2 is responsible for lipid transportation and alteration in the levels of apoA2 isoforms might be associated with hyperlipidemia. Currently we do not have data regarding lipid levels in the blood stream of the PDAC patients and controls. However, investigation of the correlation between the alteration of apoA2 isoforms and high-density lipoprotein, and hyperlipidemia are important topics for future studies.

### Establishment of a novel ELISA for measurement of the concentration of apoA2 isoforms, & confirmation of the clinical utility of ELISA of apoA2 isoforms

Although we previously reported the development of a novel and sophisticated oMALDI-qQTOF-MS method for the semiquantitative assessment of the level of apoA2 isoforms in the blood, several factors impeded the clinical application of a MALDI-TOF-MS-based method for the measurement of apoA2 [35]. One factor is because an MS-based method is difficult to use as a standardized measurement system. Another factor is that absolute quantification of molecules by MALDI-MS has not been established in a clinical setting. We therefore developed novel sandwich ELISAs for the measurement of apoA2 isoforms in clinical samples. We first established specific antibodies with high affinities for apoA2-AT/AT and apoA2-ATQ/ATQ, and we then used these antibodies to generate novel ELISA kits for measurement of apoA2-ATQ/ATQ and apoA2-AT/AT. In addition, we devised a method by which the concentration of apoA2-ATQ/AT could be calculated. The formula that we used to calculate the concentration of apoA2-ATQ/AT was the following: apoA2-ATQ/AT (μg/ml) = 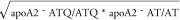 [36].

**Figure 2.**
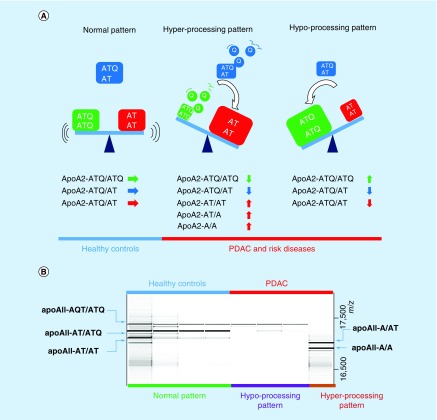
**Unique processing patterns of the C-terminal ends of the apoA2 homodimer in pancreatic ductal adenocarcinoma and precancerous lesions of PDAC.** **(A)** Normal, hypo-processing and hyper-processing patterns; and **(B)** representative gel-mobility image of MS spectra. **(A)** The normal type of apoA2 isoforms were mainly distributed in healthy controls. However, the hypo-processing pattern, in which apoA2-ATQ/ATQ was predominantly expressed, and the hyper-processing pattern, in which apoA2-AT/AT was predominantly expressed were detected in PDAC or its risk diseases. In both the hyper- and hypo-processing patterns, because apoA2-ATQ/ATQ or apoA2-AT/AT was increased, apoA2-ATQ/AT consequently decreased in PDAC [36]. **(B)** MALDI-MS spectra were converted to gel-mobility images (16,500–17,500 m/z). The expression patterns were different in each case. Top blue: healthy controls; top red: PDAC; bottom green: normal pattern; bottom purple: hypo-processing pattern; bottom brown: hyper-processing pattern [36]. MS: Mass spectrometry; PDAC: Pancreatic ductal adenocarcinoma.

**Figure 3.**
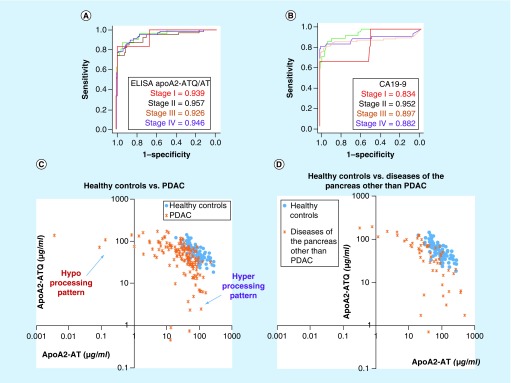
**ROC and AUC values of apoA2-ATQ/AT and CA19–9.** 2D scatter graph of apoA2-ATQ/ATQ and apoA2-AT/AT in the multi-institutions study. **(A & B)** ROC curves and AUC values of apoA2 and CA19–9 to distinguish patients with PDAC from healthy controls. The patients with PDAC were classified into clinical stages according to the Union for International Cancer Control (red line, stage I; green line, stage II, orange line, stage III and purple line, stage IV) [36].**(C & D)** 2D scatter graphs of apoA2-ATQ/ATQ (y-axis) and apoA2-AT/AT (x-axis) in PDAC (orange letter Xs) versus healthy controls (blue circles) **(C)** and diseases of the pancreas other than PDAC (orange letter Xs) versus healthy controls (blue circles) **(D)**. The representative hyper- or hypo-processing pattern was recognized in PDAC and risk diseases of pancreatic cancer [ 36]. PDAC: Pancreatic ductal adenocarcinoma; ROC: Receiver-operating characteristic.

We then measured the apoA2 isoforms again in plasma samples that were collected by seven Japanese medical institutions, using ELISA. Interestingly, when we checked the distribution of the plasma concentration of apoA2-ATQ/ATQ, which is a heavy isoform (MW, 17,380 Da), and that of apoA2-AT/AT, which is a light isoform (MW, 17,124 Da), in patients with PDAC, apoA2-ATQ/ATQ and apoA2-AT/AT showed reciprocal distributions in pancreatic cancer in comparison with healthy controls. Thus, two processing patterns of the C-terminal end of apoA2 are detected in the plasma of PDAC: one is the hyper-processing pattern, in which the light isoform apoA2-AT/AT is predominantly observed, and the second is the hypo-processing pattern in which the heavy isoform is predominantly observed (Figure 2). Neither processing pattern was detected in healthy controls. These data indicate that apoA2-ATQ/AT, which is intermediate between the heavy and light isoforms, also decreases in the plasma of PDAC compared with the healthy controls (Figure 2). A significant reduction in apoA2-ATQ/AT was detected in the plasma of any stage of PDAC in comparison with healthy controls. AUC values of apoA2-ATQ/AT to distinguish PDAC from healthy controls are shown in Figure 3A. AUC values to distinguish stage I, II, III and V were 0.939, 0.957, 0.926 and 0.946, respectively. AUC values of ELISA kits of apoA2 isoforms were higher than those of CA19–9 in any stage of PDAC (Figure 3A & B). A significant reduction in apoA2 was detected not only in PDAC but also in risk diseases of PDAC such as chronic pancreatitis, IPMN and others. The hyper- and hypo-processing patterns of apoA2 isoforms were also detected in the risk diseases of PDAC. However, although slight reductions in apoA2-ATQ/AT were detected in other gastroenterological tumors but not in pancreatic cancer, interestingly, hyper- and hypo-processing patterns were never observed in other gastroenterological diseases (Figure 3C & D). We consider that the hyper- and hypo-processing patterns might be phenomena that are unique to pancreatic diseases. The hyper- and hypo-processing might be explained by the activity of exopeptidases that are released from the pancreas into the blood stream with PDAC and other pancreatic disorders [36].

### Confirmation of the clinical utility of apoA2 isoforms measured using ELISA by the NCI EDRN

The Early Detection Research Network (EDRN), an initiative of the National Cancer Institute, is a consortium of institutions with the goal of accelerating the translation of biomarker information into clinical application for the early detection of cancer [63]. The objectives of the EDRN include the development and testing of promising biomarkers or technologies for early detection of cancer and the evaluation of promising, analytically proven biomarkers or technologies. The EDRN has reference sets available for validating promising biomarkers for the early detection of several cancers [32].

To independently confirm the clinical utility of the ELISA kits for measurement of apoA2 isoforms, NCI EDRN evaluated the ELISA kits of apoA2 isoforms with a pancreatic reference set that was collected by NCI EDRN [36]. The pancreatic cancer reference set consisting of healthy controls (n = 61), chronic pancreatitis (n = 62), acute benign biliary duct (n =31) and pancreatic cancer (stage IA/IB/IIA, n = 55; stage IIB, n = 42) was blindly measured in Japan, and the ELISA data were independently analyzed in the data management center of NCI EDRN.

When the concentration of apoA2-ATQ/AT in the reference set was compared with that in the sera of healthy controls, significant reductions in apoA2-ATQ/AT were detected in PDAC (stage IA/IB/IIA or stage IIB). The AUC value of apoA2-ATQ/AT to distinguish patients with PDAC from healthy controls was 0.809. The AUC of apoA2-ATQ/AT was higher than that of CA19-9. In addition, a significant reduction in apoA2-ATQ/AT was also detected in chronic pancreatitis, which is considered as a risk disease of PDAC.

Moreover the combination assay of CA19-9 and apoA2-ATQ/AT significantly increased the AUC value to distinguish patients with stage I/II from healthy controls (0.879) in comparison with the AUC value for CA19-9 alone (0.783). The improvement in the AUC of the combination assay of apoA2-ATQ/AT with CA19–9 was 0.098 in contrast with the AUC of CA19-9 alone (95% CI: 0.04–0.169) [36].

## Conclusion

ApoA2 isoforms are a potential biomarker for detecting patients with pancreatic cancer and the risk diseases of PDAC. It might be possible to use ApoA2 isoform measurement to enrich the general population for individuals at high risk for PDAC. The alteration of the C-terminal amino acids of the apoA2 homodimer that is seen in PDAC might be associated with the exocrine function of the pancreas.

Although numerous biomarkers have been identified by 'omics studies for the early detection of PDAC, detailed validation studies should be designed before applying these potential biomarkers to a clinical setting. The best way to confirm the claims of those interesting reports is through independent validation studies performed by other research groups or through the NCI EDRN.

## Future perspective

### The possibility of screening & risk stratification of pancreatic cancer by using a blood test of apoA2 isoforms

Screening for PDAC in high-risk individuals, such as subjects with a background of hereditary factors, adult onset diabetes, patients with cystic diseases that are considered as precancerous lesions (IPMN and MCN) and patients with a history of a genetic syndrome associated with pancreatic cancer, will continue in the next few years. Promising results are already being reported. Recently, the outcomes of long-term prospective follow-up studies were reported from European expert centers. They concluded that surveillance of CDNK2A mutation carriers was relatively successful, detecting most PDACs at a resectable stage [64].

As described here, apoA2 isoforms may not only be useful as a plasma biomarker to distinguish PDAC, but they also have the potential to distinguish diseases in asymptomatic individuals that have a risk of developing PDAC. Measurement of apoA2 isoforms for detecting stage I/II and the risk diseases of PDAC is a biomarker that can be detected using a blood test. Therefore, apoA2 is a potential biomarker for filtering of the general population to enrich for individuals at high risk of pancreatic cancer.

Proposed biomarkers for early detection of pancreatic cancer and risk diseases of PDAC will have to be confirmed using prospective validation studies that are designed for the surveillance of individuals at risk for pancreatic cancer. In addition, if such a biomarker can detect individuals at risk in the general population, it would have the potential to identify subjects who should undergo invasive modalities to detect resectable pancreatic cancers after first filtering the population using a noninvasive and inexpensive examination.

To date, the final diagnosis of pancreatic cancer has been made using imaging and/or pathological findings obtained from fine-needle aspiration biopsy. The final diagnosis of pancreatic cancer is not based only on the result of a blood test. The presence of a biomarker that can be assayed by an inexpensive blood test allows screening for the early stage of pancreatic cancer and the risk diseases including IPMN, MCN and chronic pancreatitis. A first screening should therefore be performed in asymptomatic individuals using this inexpensive biomarker and, after identification of individuals at risk, the more expensive imaging modality should be used to diagnose the pancreatic cancer.

The concept is similar to that of the fecal occult blood test for screening of colorectal cancer. Thus, although the fecal occult blood test for colorectal cancer screening is not an IVD with high sensitivity and specificity, screening using this fecal occult blood test has the effect of reducing mortality due to colorectal cancer.

The mortality of pancreatic cancer is one of the worst in the world of diseases of solid malignant tumors. Future study involving international collaboration for early detection of pancreatic cancer is important in order to decrease the mortality rate of pancreatic cancer. Future development of an early detection program for pancreatic cancer through international collaboration is needed.

**Executive summary**

- To decrease the mortality of pancreatic cancer, the development of screening methods for early stages of pancreatic cancer is urgently needed.
- Due to the low incidence of pancreatic cancer, it is generally considered that the screening of the general population for pancreatic cancer using invasive and expensive modalities is difficult taking into consideration the cost–effectiveness. Combination strategies with biomarkers and imaging are attractive for pancreatic cancer screening.

**Existing conventional biomarkers for the detection of pancreatic cancer using a blood test**

- There are no existing conventional biomarkers that are recommended by the American Society of Clinical Oncology for the early detection of pancreatic cancer.
- In the last decade, some clinically attractive biomarkers for early detection of pancreatic cancer using blood tests have been identified by omics studies. To accelerate the clinical development of new biomarkers, it is important to rapidly evaluate the clinical usefulness of potential biomarkers. The best method for confirming the claims of these interesting reports is to have independent validation studies performed by other research groups or by the National Cancer Institute Early Detection Research Network.

**Possible detection of the early stage of pancreatic cancer & of risk diseases by apolipoprotein A2 isoforms**

- Unique alteration of processing patterns in the apoA2 homodimer was detected in the blood of patients with pancreatic cancer and its risk diseases. The potential usefulness of apoA2 isoforms as a plasma biomarker for the screening and risk stratification of pancreatic has been validated by Japanese study groups, a German study and the confirmation program of the National Cancer Institute Early Detection Research Network.

**Future perspective**

- The mortality of pancreatic cancer is one of the worst in the world of diseases of solid malignant tumors. Future study involving international collaboration for early detection of pancreatic cancer is important in order to decrease the mortality rate of pancreatic cancer. Development of an early detection program for pancreatic cancer through international collaboration is needed.

## References

[1] Hori M, Matsuda T, Shibata A, Katanoda K, Sobue T, Nishimoto H (2015). Cancer incidence and incidence rates in Japan in 2009: a study of 32 population-based cancer registries for the Monitoring of Cancer Incidence in Japan (MCIJ) project. *Jpn. J. Clin. Oncol.*.

[2] Siegel R, Naishadham D, Jemal A (2013). Cancer statistics: 2013. *CA Cancer J. Clin.*.

[3] KapWeb Survival statistics of Japanese association of Clinical Cancer Centers. http://www.kapweb.chiba-cancer-registry.org/?lang=en.

[4] Modolell I, Guarner L, Malagelada JR (1999). Vagaries of clinical presentation of pancreatic and biliary tract cancer. *Ann. Oncol.*.

[5] Ducreux M, Cuhna AS, Caramella C (2015). Cancer of the pancreas: ESMO Clinical Practice Guidelines for diagnosis: treatment and follow-up. *Ann. Oncol.*.

[6] Porta M, Fabregat X, Malats N (2005). Exocrine pancreatic cancer: symptoms at presentation and their relation to tumour site and stage. *Clin. Transl. Oncol.*.

[7] Ryan DP, Hong TS, Bardeesy N (2014). Pancreatic adenocarcinoma. *N. Engl. J. Med.*.

[8] Chari ST, Kelly K, Hollingsworth MA (2015). Early detection of sporadic pancreatic cancer: summative review. *Pancreas*.

[9] Sohn TA, Yeo CJ, Cameron JL (2000). Resected adenocarcinoma of the pancreas-616 patients: results, outcomes, and prognostic indicators. *J. Gastrointest. Surg.*.

[10] Yachida S, Jones S, Bozic I (2010). Distant metastasis occurs late during the genetic evolution of pancreatic cancer. *Nature*.

[11] Chari ST, Leibson CL, Rabe KG, Ransom J, De Andrade M, Petersen GM (2005). Probability of pancreatic cancer following diabetes: a population-based study. *Gastroenterology*.

[12] Pannala R, Basu A, Petersen GM, Chari ST (2009). New-onset diabetes: a potential clue to the early diagnosis of pancreatic cancer. *Lancet Oncol.*.

[13] Chari ST (2007). Detecting early pancreatic cancer: problems and prospects. *Semin. Oncol.*.

[14] Klein AP, Brune KA, Petersen GM (2004). Prospective risk of pancreatic cancer in familial pancreatic cancer kindreds. *Cancer Res.*.

[15] Brune KA, Lau B, Palmisano E (2010). Importance of age of onset in pancreatic cancer kindreds. *J. Natl Cancer Inst.*.

[16] Jacobs EJ, Chanock SJ, Fuchs CS (2010). Family history of cancer and risk of pancreatic cancer: a pooled analysis from the Pancreatic Cancer Cohort Consortium (PanScan). *Int. J. Cancer*.

[17] Klein AP (2013). Identifying people at a high risk of developing pancreatic cancer. *Nat. Rev. Cancer*.

[18] Kamisawa T, Wood LD, Itoi T, Takaori K (2016). Pancreatic cancer. *Lancet*.

[19] Iodice S, Gandini S, Maisonneuve P, Lowenfels AB (2008). Tobacco and the risk of pancreatic cancer: a review and meta-analysis. *Langenbecks Arch. Surg.*.

[20] Arslan A, Helzlsouer KJ, Kooperberg C (2010). Anthropometric measures, body mass index, and pancreatic cancer: a pooled analysis from the Pancreatic Cancer Cohort Consortium (PanScan). *Arch. Intern. Med.*.

[21] Hassan M, Bondy ML, Wolff RA (2007). Risk factors for pancreatic cancer: case-control study. *Am. J. Gastroenterol.*.

[22] Larsson SC, Wolk A (2012). Red and processed meat consumption and risk of pancreatic cancer: meta-analysis of prospective studies. *Br. J. Cancer*.

[23] Yeo TP (2015). Demographics, epidemiology, and inheritance of pancreatic ductal adenocarcinoma. *Semin. Oncol.*.

[24] Maisonneuve P, Lowenfels AB (2015). Risk factors for pancreatic cancer: a summary review of meta-analytical studies. *Int. J. Epidemiol.*.

[25] Rebours V, Boutron-Ruault MC, Schnee M (2008). Risk of pancreatic adenocarcinoma in patients with hereditary pancreatitis: a national exhaustive series. *Am. J. Gastroenterol.*.

[26] Iqbal J, Ragone A, Lubinski J (2012). The incidence of pancreatic cancer in BRCA1 and BRCA2 mutation carriers. *Br. J. Cancer*.

[27] Jones S, Hruban RH, Kamiyama M (2009). Exomic sequencing identifies PALB2 as a pancreatic cancer susceptibility gene. *Science*.

[28] Giardiello FM, Brensinger JD, Tersmette AC (2000). Very high risk of cancer in familial Peutz–Jeghers syndrome. *Gastroenterology*.

[29] Hruban RH, Goggins M, Parsons J, Kern SE (2000). Progression model for pancreatic cancer. *Clin. Cancer Res.*.

[30] Hruban RH, Takaori K, Klimstra DS (2004). An illustrated consensus on the classification of pancreatic intraepithelial neoplasia and intraductal papillary mucinous neoplasms. *Am. J. Surg. Pathol.*.

[31] Zamboni G, Hirabayashi K, Castelli P, Lennon AM (2013). Precancerous lesions of the pancreas. *Best Pract. Res. Clin. Gastroenterol.*.

[32] Srivastava S (2013). The early detection research network: 10 year outlook. *Clin. Chem.*.

[33] Feng Z, Kagan J, Pepe M (2013). The Early Detection Research Network’s Specimen reference sets: paving the way for rapid evaluation of potential biomarkers. *Clin. Chem.*.

[34] Honda K, Hayashida Y, Umaki T (2005). Possible detection of pancreatic cancer by plasma protein profiling. *Cancer Res.*.

[35] Honda K, Okusaka T, Felix K (2012). Altered plasma apolipoprotein modifications in patients with pancreatic cancer: protein characterization and multi-institutional validation. *PLoS ONE*.

[36] Honda K, Kobayashi M, Okusaka T (2015). Plasma biomarker for detection of early stage pancreatic cancer and risk factors for pancreatic malignancy using antibodies for apolipoprotein-AII isoforms. *Sci. Rep.*.

[37] Poruk KE, Gay DZ, Brown K (2013). The clinical utility of CA 19–9 in pancreatic adenocarcinoma: diagnostic and prognostic updates. *Curr. Mol. Med.*.

[38] Ferrone CR, Finkelstein DM, Thayer SP, Muzikansky A, Fernandez-Delcastillo C, Warshaw AL (2006). Perioperative CA19–9 levels can predict stage and survival in patients with resectable pancreatic adenocarcinoma. *J. Clin. Oncol.*.

[39] Takasaki H, Uchida E, Tempero MA, Burnett DA, Metzgar RS, Pour PM (1988). Correlative study on expression of CA 19–9 and DU-PAN-2 in tumor tissue and in serum of pancreatic cancer patients. *Cancer Res.*.

[40] Tempero MA, Uchida E, Takasaki H, Burnett DA, Steplewski Z, Pour PM (1987). Relationship of carbohydrate antigen 19–9 and Lewis antigens in pancreatic cancer. *Cancer Res.*.

[41] Locker GY, Hamilton S, Harris J (2006). ASCO 2006 update of recommendations for the use of tumor markers in gastrointestinal cancer. *J. Clin. Oncol.*.

[42] Kim JE, Lee KT, Lee JK, Paik SW, Rhee JC, Choi KW (2004). Clinical usefulness of carbohydrate antigen 19–9 as a screening test for pancreatic cancer in an asymptomatic population. *J. Gastroenterol. Hepatol.*.

[43] Goonetilleke KS, Siriwardena AK (2007). Systematic review of carbohydrate antigen (CA 19–9) as a biochemical marker in the diagnosis of pancreatic cancer. *Eur. J. Surg. Oncol.*.

[44] Kawa S, Oguchi H, Kobayashi T (1991). Elevated serum levels of Dupan-2 in pancreatic cancer patients negative for Lewis blood group phenotype. *Br. J. Cancer*.

[45] Frena A (2001). SPan-1 and exocrine pancreatic carcinoma. The clinical role of a new tumor marker. *Int. J. Biol. Markers*.

[46] Sugano K, Ohkura H, Maruyama T (1988). Sandwich radioimmunometric assay with murine monoclonal antibody, NCC-ST-439, for serological diagnosis of human cancers. *Jpn. J. Cancer Res.*.

[47] Watanabe M, Hirohashi S, Shimosato Y (1985). Carbohydrate antigen defined by a monoclonal antibody raised against a gastric cancer xenograft. *Jpn. J. Cancer Res.*.

[48] Koopmann J, Fedarko NS, Jain A (2004). Evaluation of osteopontin as biomarker for pancreatic adenocarcinoma. *Cancer Epidemiol. Biomarkers Prev.*.

[49] Koopmann J, Buckhaults P, Brown DA (2004). Serum macrophage inhibitory cytokine 1 as a marker of pancreatic and other periampullary cancers. *Clin. Cancer Res.*.

[50] Ono M, Matsubara J, Honda K (2009). Prolyl 4-hydroxylation of alpha-fibrinogen: a novel protein modification revealed by plasma proteomics. *J. Biol. Chem.*.

[51] Kuhlmann KF, Van Till JW, Boermeester MA (2007). Evaluation of matrix metalloproteinase 7 in plasma and pancreatic juice as a biomarker for pancreatic cancer. *Cancer Epidemiol. Biomarkers Prev.*.

[52] Kobayashi T, Nishiumi S, Ikeda A (2013). A novel serum metabolomics-based diagnostic approach to pancreatic cancer. *Cancer Epidemiol. Biomarkers Prev.*.

[53] Melo SA, Luecke LB, Kahlert C (2015). Glypican-1 identifies cancer exosomes and detects early pancreatic cancer. *Nature*.

[54] Herreros-Villanueva M, Bujanda L (2016). Glypican-1 in exosomes as biomarker for early detection of pancreatic cancer. *Ann. Transl. Med.*.

[55] Kojima M, Sudo H, Kawauchi J (2015). MicroRNA markers for the diagnosis of pancreatic and biliary-tract cancers. *PLoS ONE*.

[56] Diamandis EP, Plebani M (2016). Glypican-1 as a highly sensitive and specific pancreatic cancer biomarker. *Clin. Chem. Lab. Med.*.

[57] Honda K, Ono M, Shitashige M (2013). Proteomic approaches to the discovery of cancer biomarkers for early detection and personalized medicine. *Jpn. J. Clin. Oncol.*.

[58] Ehmann M, Felix K, Hartmann D (2007). Identification of potential markers for the detection of pancreatic cancer through comparative serum protein expression profiling. *Pancreas*.

[59] Xue A, Scarlett CJ, Chung L (2010). Discovery of serum biomarkers for pancreatic adenocarcinoma using proteomic analysis. *Br. J. Cancer*.

[60] Blanco-Vaca F, Via DP, Yang CY, Massey JB, Pownall HJ (1992). Characterization of disulfide-linked heterodimers containing apolipoprotein D in human plasma lipoproteins. *J. Lipid Res.*.

[61] Pankhurst G, Wang XL, Wilcken DE (2003). Characterization of specifically oxidized apolipoproteins in mildly oxidized high density lipoprotein. *J. Lipid Res.*.

[62] Smith LE, Yang J, Goodman L (2012). High yield expression and purification of recombinant human apolipoprotein A-II in *Escherichia coli*. *J. Lipid Res.*.

[63] National Cancer Institute Early Detection Research Network. http://www.edrn.nci.nih.gov/.

[64] Vasen H, Ibrahim I, Ponce CG (2016). Benefit of surveillance for pancreatic cancer in high-risk individuals: outcome of long-term prospective follow-up studies from three European expert centers. *J. Clin. Oncol.*.

